# LEARNING ABOUT THE PROCESS OF REVISION OF POLICIES AROUND INNOVATIVE CARE MODELS AT THE VHA

**DOI:** 10.1093/geroni/igae098.0200

**Published:** 2024-12-31

**Authors:** Quratulain Syed

**Affiliations:** Joseph Maxwell Cleland Atlanta VA Medical Center, Atlanta, Georgia, United States

## Abstract

Having practiced as a geriatrician in underserved neighborhoods and post-acute care in the United States, I have witnessed older adults and individuals with multimorbidity facing the brunt of health policies that favor the healthy and the well-resourced. These observations have formulated my vision for age friendly health policies, and the need for front line clinicians to get a seat at the health policy table and influence the policies. The VA track of the Health and Aging Policy Fellows’ Program is an opportunity for professionals involved in healthcare and research focused on older Veterans to nurture skill necessary for development and implementation of health policies that impact older Veterans. During the session, I will discuss my placement at the Veterans Health Administration Central Office with the Home & Community Based Programs. This opportunity has allowed me to learn about the process of updating and revision of policies and directives around innovative care models with demonstrated clinical outcomes.

